# Reducing Delays, Improving Flow: The Importance of a Dedicated Discharge Coordinator in Hospital Discharge Planning

**DOI:** 10.7759/cureus.85879

**Published:** 2025-06-12

**Authors:** George Bechir, Angelina Bechir

**Affiliations:** 1 Hospital Medicine, Franciscan Health Munster, Munster, USA; 2 Genetics and Biochemistry, Clemson University, Clemson, USA

**Keywords:** care transitions, discharge coordinator, discharge planning, hospital discharge, hospital efficiency, length of stay, patient flow, patient throughput, readmission reduction, same-day discharge

## Abstract

Hospital discharge remains one of the most vulnerable stages of inpatient care, frequently delayed not by clinical need but by fragmented coordination of tasks such as imaging, consults, documentation, and transportation. These bottlenecks contribute to prolonged length of stay (LOS), emergency department crowding, higher healthcare costs, and decreased patient satisfaction. In this article, we examine the critical role of a dedicated discharge coordinator - a nurse, nurse practitioner, or case manager - who is tasked solely with tracking and resolving same-day discharge barriers in real time. Drawing on findings from 20 peer-reviewed studies, we synthesize evidence demonstrating that such centralized oversight reduces LOS (often by 0.5 to 1 day), improves patient throughput, decreases readmission rates, and enhances communication between care teams. We explore various coordination models, ranging from unit-based facilitators to electronic health record (EHR)-integrated tracking tools, and present real-world examples where minor misalignments in timing, such as delayed dialysis or unscheduled imaging, led to avoidable hospital days, reinforcing the need for structured and accountable discharge management. The discharge coordinator acts as the operational "traffic controller" of inpatient flow, ensuring timely, safe, and coordinated transitions of care. In today’s strained hospital systems, this role is essential for maintaining safety, efficiency, and capacity.

## Editorial

Introduction

Hospital discharge is often viewed as a pivotal transition in a patient’s care journey - one that significantly influences outcomes after hospitalization. While the clinical aspects of inpatient care are typically well-structured, guided by diagnostic algorithms, treatment protocols, and interdisciplinary rounds, discharge planning frequently unfolds with far less coordination [1]. Each day, patients deemed medically ready for discharge remain hospitalized due to administrative or logistical barriers, not clinical concerns. These delays are widespread and costly, increasing the risk of hospital-acquired complications, consuming limited bed capacity, prolonging emergency department (ED) boarding, and disrupting continuity of care [1,2]. From a systems perspective, they also reduce throughput and elevate costs. Despite the involvement of hospitalists, nurses, case managers, consultants, and ancillary services, no single team member is typically responsible for overseeing and aligning all discharge-related tasks in real time [1,2]. In many cases, these delays arise simply from a lack of coordination, missed follow-through, or unclear accountability - issues that a dedicated discharge coordinator is specifically designed to resolve.

In most hospitals, discharge is a shared responsibility, but not a clearly owned one. A hospitalist may determine readiness, a nurse may prepare the patient, a case manager may arrange services, and a specialist may finalize recommendations. Yet without centralized, accountable coordination, tasks such as imaging, dialysis, prescription reconciliation, transportation, and placement often occur too late in the day or not at all [1,3]. This article explores a practical and scalable solution: the implementation of a dedicated discharge coordinator, whether a nurse, nurse practitioner, or case manager, whose sole responsibility is to track and resolve pending discharge tasks for patients scheduled to leave that day, serving as the operational “traffic light” in a congested hospital intersection, ensuring that all care team members and departments are aligned to facilitate safe, same-day discharge [1,4]. Dunn and Rogers described an innovative model where a pediatric nurse practitioner served as a discharge facilitator, focusing solely on preparing families for discharge and ensuring all tasks were complete, improving care continuity and family readiness while streamlining the discharge process [5]. We draw upon 20 peer-reviewed studies that evaluate discharge coordination across various clinical settings, patient populations, and intervention models. Our goal is to highlight the measurable impact of assigning discharge ownership to a single, dedicated role and to argue that hospitals can no longer afford to treat discharge as an afterthought; they must approach it as a critical phase of care requiring proactive, real-time management [6,7]. Importantly, the discharge coordinator does not determine medical readiness for discharge; that responsibility remains solely with the treating physician.

The discharge process in hospitals has long been a source of inefficiency, often lacking the structure and accountability needed for timely patient transitions. A consistent theme in the literature is the benefit of assigning a dedicated discharge coordinator - a nurse, nurse practitioner, or case manager - responsible for resolving discharge-related tasks in real time [1,2]. Across diverse hospital settings, patient populations, and intervention models, centralized oversight through a discharge coordinator has been associated with improved throughput, reduced length of stay (LOS), and lower readmission rates, demonstrating the operational and clinical value of this role [1-10]. Importantly, this role also facilitates clearer communication among care teams by serving as a central point of contact and ensuring that updates, orders, and task completions are communicated promptly and efficiently. As several studies show, such coordination not only expedites discharge but also supports safer transitions by minimizing errors, redundancy, and missed steps that commonly occur when no one person is accountable for managing same-day discharge logistics [1-10].

Unit-based, team-based, and facilitator roles

One of the most illustrative studies comes from Petitgout, who introduced a unit-based discharge coordinator in a pediatric inpatient unit responsible for attending daily rounds, identifying patients eligible for same-day discharge, and proactively resolving remaining barriers - whether educational, procedural, or logistical - resulting in a measurable increase in patient satisfaction, reduction in discharge-related safety events, and decline in LOS, demonstrating the operational value of the role [1]. Building on this, Fatani et al. applied a team-based model in neurology inpatient departments, establishing a dedicated discharge planning team composed of physicians, nurses, and social workers. The presence of this team led to significantly earlier discharges and reduced readmissions, suggesting that even without a single coordinator, consolidating discharge responsibility within a defined group yields operational and clinical benefits [2]. Houghton et al. found that a discharge coordinator improved post-discharge communication and reduced issues with follow-up, further enhancing care continuity [3]. Together, these models clearly demonstrate the vital role of the discharge coordinator as the “traffic controller” of inpatient flow, effectively ensuring timely, safe, and efficient patient transitions. Their presence is essential to coordinating complex discharge processes and optimizing patient throughput.

Technology-based and interdisciplinary planning tools

Kutz et al. evaluated a technologically advanced intervention using an electronic health record (EHR) tool designed for interprofessional discharge planning, which, though not a person, functioned similarly by centralizing task tracking and communication across disciplines. This resulted in a statistically significant reduction in LOS among patients with multimorbidity, reinforcing the idea that centralization - whether human or digital - matters [4]. Dunn and Rogers described an innovative model where a pediatric nurse practitioner served as a discharge facilitator, focusing solely on preparing families for discharge and ensuring all tasks were complete, improving care continuity and family readiness while streamlining the discharge process [5]. Hunt-O’Connor et al. found that a dedicated discharge coordinator improved the quality of hospital discharge, and DeMaio et al. implemented the PROPEL Discharge initiative - embedding a facilitator into interdisciplinary rounds who monitored labs, imaging, consults, and placement status in real time, resulting in faster discharges, improved communication, and fewer missed opportunities to get patients home [6,7]. These findings unequivocally establish the discharge coordinator - whether a human professional or a digital system - as the indispensable operational “traffic controller” of inpatient flow, playing a critical role in eliminating bottlenecks, expediting discharges, and ensuring seamless, timely, and efficient care transitions across complex hospital environments.

Dedicated units and discharge efficiency

Operational models at the unit level further reinforce these benefits. Curle and Maduro described a dedicated discharge unit staffed by nurses who only handled final-day discharge logistics, which reduced LOS and increased ED capacity by accelerating patient flow at the back end of care [8]. Salinkar et al. evaluated a nurse-led discharge liaison in a community hospital, reporting improved task completion, greater care team collaboration, and reduced delays. The intervention improved discharge times by 32% and reduced bed turnaround time by 18% [9]. Destino et al. reported that implementing a multimodal intervention increased early discharges from 8.8% to 15.8% and significantly reduced ED wait times (from 221 to 133 minutes) and post-anesthesia care unit (PACU) delays (from 56 to 36 minutes), highlighting the impact of coordinated discharge efforts [10]. Together, these findings underscore the discharge coordinator’s pivotal role in orchestrating efficient patient flow, demonstrating that centralized, real-time oversight is not just beneficial but essential for sustaining hospital throughput, patient safety, and overall system capacity.

Discharge timing, readmission risk, and continuity of care

Several studies have explored the relationship between discharge timing and downstream outcomes. Misky et al. found that delays in post-discharge follow-up or poor coordination were directly tied to higher readmission rates. Hansen et al. reinforced this in a systematic review of 30-day rehospitalization interventions, showing that discharge coordination was a central component in successful models [11,12]. Balaban et al. found that assigning a patient navigator to coordinate post-discharge care for high-risk patients resulted in fewer readmissions, while Lien et al. examined how hospital ownership models influence treatment quality and efficiency in stroke and cardiac care, suggesting that institutional factors can impact patient outcomes and care processes [13,14]. McMurray and Stewart reviewed the evidence showing that nurse-led, multidisciplinary interventions can improve outcomes and reduce hospitalizations in chronic heart failure patients, emphasizing factors like improved patient understanding, adherence, and discharge planning. Weinberger et al. also found that increased access to primary care can reduce hospital readmissions [15,16]. van Walraven et al. showed that a significant proportion of urgent readmissions could have been prevented through better coordination at discharge, and Kripalani et al. identified poor communication between inpatient and outpatient teams as a key barrier to safe transitions, arguing for a dedicated person to manage these gaps [17,18]. Taken together, these studies underscore that without a clearly designated discharge coordinator, the transition of care remains fragmented and vulnerable. By serving as the operational hub for communication, logistics, and follow-up planning, the discharge coordinator is not just helpful but essential for ensuring safe, timely, and effective transitions that reduce readmissions and improve overall patient outcomes.

Broader health system factors and specialty populations

Zhu et al. conducted a systematic review and meta-analysis demonstrating that nurse-led early discharge planning programs significantly improved outcomes for hospitalized patients with chronic diseases or rehabilitation needs. These programs were associated with reduced readmissions and improved continuity of care [19]. Similarly, Feltner et al., in a comprehensive review published in Annals of Internal Medicine, concluded that transitional care interventions, particularly those that incorporated dedicated discharge support, were effective in lowering readmission rates for patients with heart failure while enhancing care transitions [20]. These high-quality studies reinforce the discharge coordinator’s pivotal role in safeguarding post-hospital outcomes. Far from being an optional support, the discharge coordinator is a vital safeguard for continuity, efficiency, and patient safety, especially in populations where poor coordination can have lasting clinical consequences.

Discussion

While the literature clearly illustrates the value of discharge coordination, the true power of this role becomes even more evident when examined through the lens of daily hospital realities, where minor delays, left unmanaged, spiral into costly system-wide inefficiencies. Each morning, care teams identify patients as “ready for discharge,” yet many remain hospitalized by day’s end, not because their condition worsens, but because essential discharge tasks are delayed or uncoordinated, such as unscheduled imaging, overlapping dialysis and transport, or incomplete documentation [6,12,20]. These delays are operational failures, not clinical ones, and while multiple professionals contribute to discharge planning, the absence of a single person responsible for coordinating tasks often leads to inefficiencies. The discharge coordinator fills this gap by focusing solely on patients expected to leave that day, ensuring all components, labs, imaging, consults, prescriptions, and placement are addressed early and proactively [6,12]. This role does not replace others; it synchronizes them. The discharge coordinator does not merely follow a checklist; they identify and escalate delays before they become barriers, acting as a "traffic light" at a busy intersection, ensuring departments do not operate in conflicting or redundant directions [6]. Real-world examples illustrate the need, such as one patient missing magnetic resonance imaging (MRI) because dialysis was occurring when radiology called, and another’s discharge being postponed because dialysis ended too late for a skilled nursing facility to accept the transfer. These delays are not rare; they occur daily, and without coordination, the patient stays another night [6,12]. Studies by Hunt O’Connor et al., Hansen et al., and Feltner et al. reinforce this role’s importance, showing that structured discharge coordination reduces both LOS and readmissions. The model can vary, from embedded nurse practitioners to discharge-specific units or digital tools, but the common denominator is clear ownership of the discharge process [6,12,20]. As hospitals face rising demands, ED boarding, and staff burnout, the discharge coordinator is a high-impact, scalable solution [6,12,20].

Conclusion

Discharge is not merely the end of a hospital stay; it is a critical transition that reflects the success or failure of the entire hospitalization. Yet across health systems, this transition remains fragmented, delayed, and too often overlooked, resulting in preventable delays, prolonged LOS, and disrupted continuity of care. While clinical care is tightly managed, discharge is frequently left to chance. A dedicated discharge coordinator, whether a single clinician, a team, or a technology-driven solution, provides the accountability and real-time oversight needed to streamline tasks, reduce readmissions, and improve patient flow.

## References

[1] Petitgout JM (2015). Implementation and evaluation of a unit-based discharge coordinator to improve the patient discharge experience. J Pediatr Health Care.

[2] Fatani A, Alzebaidi S, Alghaythee HK (2025). The role of the discharge planning team on the length of hospital stay and readmission in patients with neurological conditions: a single-center retrospective study. Healthcare (Basel).

[3] Houghton A, Bowling A, Clarke KD, Hopkins AP, Jones I (1996). Does a dedicated discharge coordinator improve the quality of hospital discharge?. Qual Health Care.

[4] Kutz A, Koch D, Haubitz S (2022). Association of interprofessional discharge planning using an electronic health record tool with hospital length of stay among patients with multimorbidity: a nonrandomized controlled trial. JAMA Netw Open.

[5] Dunn K, Rogers J (2016). Discharge facilitation: an innovative PNP role. J Pediatr Health Care.

[6] Hunt-O'Connor C, Moore Z, Patton D, Nugent L, Avsar P, O'Connor T (2021). The effect of discharge planning on length of stay and readmission rates of older adults in acute hospitals: a systematic review and meta-analysis of systematic reviews. J Nurs Manag.

[7] DeMaio J, Purdy O, Ghidini J, Menillo J, Viney R, Hogan C (2025). PROPEL discharge: an interdisciplinary throughput initiative. Jt Comm J Qual Patient Saf.

[8] Curle BW, Maduro R (2020). Discharge unit efficiency. Am Nurse J.

[9] Salinkar J, Pierce D, Khasawneh MT, Wang S (2010). Streamlining the discharge process with a dedicated discharge nurse. IISE Conf Proc.

[10] Destino L, Bennett D, Wood M, Acuna C, Goodman S, Asch SM, Platchek T (2019). Improving patient flow: analysis of an initiative to improve early discharge. J Hosp Med.

[11] Misky GJ, Wald HL, Coleman EA (2010). Post-hospitalization transitions: examining the effects of timing of primary care provider follow-up. J Hosp Med.

[12] Hansen LO, Young RS, Hinami K, Leung A, Williams MV (2011). Interventions to reduce 30-day rehospitalization: a systematic review. Ann Intern Med.

[13] Balaban RB, Galbraith AA, Burns ME, Vialle-Valentin CE, Larochelle MR, Ross-Degnan D (2015). A patient navigator intervention to reduce hospital readmissions among high-risk safety-net patients: a randomized controlled trial. J Gen Intern Med.

[14] Lien HM, Chou SY, Liu JT (2008). Hospital ownership and performance: evidence from stroke and cardiac treatment in Taiwan. J Health Econ.

[15] McMurray JJ, Stewart S (1998). Nurse led, multidisciplinary intervention in chronic heart failure. Heart.

[16] Weinberger M, Oddone EZ, Henderson WG (1996). Does increased access to primary care reduce hospital readmissions? Veterans affairs cooperative study group on primary care and hospital readmission. N Engl J Med.

[17] van Walraven C, Jennings A, Taljaard M (2011). Incidence of potentially avoidable urgent readmissions and their relation to all-cause urgent readmissions. CMAJ.

[18] Kripalani S, Jackson AT, Schnipper JL, Coleman EA (2007). Promoting effective transitions of care at hospital discharge: a review of key issues for hospitalists. J Hosp Med.

[19] Zhu QM, Liu J, Hu HY, Wang S (2015). Effectiveness of nurse-led early discharge planning programmes for hospital inpatients with chronic disease or rehabilitation needs: a systematic review and meta-analysis. J Clin Nurs.

[20] Feltner C, Jones CD, Cené CW (2014). Transitional care interventions to prevent readmissions for persons with heart failure: a systematic review and meta-analysis. Ann Intern Med.

